# Biological, Psychological, and Sociocultural Factors Contributing to the Drive for Muscularity in Weight-Training Men

**DOI:** 10.3389/fpsyg.2016.01992

**Published:** 2016-12-21

**Authors:** Catharina Schneider, Laura Rollitz, Martin Voracek, Kristina Hennig-Fast

**Affiliations:** ^1^Department of Applied Psychology: Health, Development, Enhancement, and Intervention, Faculty of Psychology, University of ViennaVienna, Austria; ^2^Department of Basic Psychological Research and Research Methods, Faculty of Psychology, University of ViennaVienna, Austria; ^3^Department of Psychiatry and Psychotherapy, Evangelisches Krankenhaus BielefeldBielefeld, Germany

**Keywords:** drive for muscularity, ideal body internalization, self-esteem, body dissatisfaction, age, fat-free mass index

## Abstract

The drive for muscularity and associated behaviors (e.g., exercising and dieting) are of growing importance for men in Western societies. In its extreme form, it can lead to body image concerns and harmful behaviors like over-exercising and the misuse of performance-enhancing substances. Therefore, investigating factors associated with the drive for muscularity, especially in vulnerable populations like bodybuilders and weight trainers can help identify potential risk and protective factors for body image problems. Using a biopsychosocial framework, the aim of the current study was to explore different factors associated with drive for muscularity in weight-training men. To this purpose, German-speaking male weight trainers (*N* = 248) completed an online survey to determine the extent to which biological, psychological, and sociocultural factors contribute to drive for muscularity and its related attitudes and behaviors. Using multiple regression models, findings showed that media ideal body internalization was the strongest positive predictor for drive for muscularity, while age (*M* = 25.9, *SD* = 7.4) held the strongest negative association with drive for muscularity. Dissatisfaction with muscularity, but not with body fat, was related to drive for muscularity. The fat-free mass index, a quantification of the actual degree of muscularity of a person, significantly predicted drive for muscularity-related behavior but not attitudes. Body-related aspects of self-esteem, but not global self-esteem, were significant negative predictors of drive for muscularity. Since internalization of media body ideals presented the highest predictive value for drive for muscularity, these findings suggest that media body ideal internalizations may be a risk factor for body image concerns in men, leading, in its most extreme form to disordered eating or muscle dysmorphia. Future research should investigate the relations between drive for muscularity, age, body composition, internalization, dissatisfaction with muscularity and body-related self-esteem using longitudinal study designs. Limitations concern the cross-sectional design of the study, self-reported body composition measures and the homogeneity of the sample.

## Introduction

Body image is a multidimensional construct which is defined by the perception of, and the attitudes about, one’s body (Cash and Brown, 1989). It refers to “how people think, feel, and behave with regard to their own physical attributes” (Muth and Cash, 1997, p. 1438) and it is influenced, amongst others, by socially prescribed body ideals (Keery et al., 2004). Similar to female body ideals growing ever thinner, the male ideal has come to be increasingly muscular over the last decades (Pope et al., 1998; Leit et al., 2001; Baghurst et al., 2006). Also, the appearance of (half-naked) male bodies in the media has grown steadily (Pope et al., 2001).

These increasingly muscular ideals have been found to influence men’s view of their own bodies. It was shown, that even a brief presentation of pictures of muscular men lead men to report a greater discrepancy between their own muscularity and the level of muscularity which they ideally wanted to possess (Leit et al., 2002). Increasing numbers of gym subscriptions, men-oriented magazines (Pope et al., 2000b) and advertisements using half-naked male bodies (Pope et al., 2001) point to the growing importance of this ideal male body image. With increasing pressure to achieve this ideal, negative effects such as body dissatisfaction or body shame are likely. Body dissatisfaction, media pressure, and negative affect are some of the factors potentially leading to muscle dysmorphia (MD), an extreme form of distorted body image (Olivardia et al., 2004; Grieve, 2007). MD refers to a pathological preoccupation of not being sufficiently large and muscular, while usually being more muscular than the average person (Pope et al., 1997). Also related to negative male body image is the misuse of performance enhancing substances, which can lead to negative physiological (Kanayama et al., 2008) and psychological (Casavant et al., 2007) outcomes.

Thus, a growing field of research is engaged in investigating factors that may have a negative influence on male body image (Ricciardelli et al., 2000; Cafri et al., 2006; Hobza et al., 2007). Next to men’s dissatisfaction with their muscularity or body fat, an important aspect associated with body image is drive for muscularity. It reflects the extent to which individuals strive to become more muscular and can be represented in attitudes (e.g., the desire for muscularity) and behaviors (e.g., weight-lifting; McCreary, 2012). Taken to the extreme, drive for muscularity can lead to MD, just as drive for thinness can reach pathological degrees resulting in eating disorders (Robert et al., 2009).

The preoccupation for lean muscularity and thus the drive for muscularity are found to be the strongest in bodybuilding and weight-training men (Hallsworth et al., 2005; Hale et al., 2010). They are also generally at a higher risk for developing a pathological engagement with their bodies than men not engaged in these kinds of sports (Blouin and Goldfield, 1995; Nieuwoudt et al., 2015). Still, factors associated with drive for muscularity in weight-training men have scarcely been investigated, but instead college and community samples have been used (e.g., McCreary, 2012).

The factors associated with the drive for muscularity can be roughly divided in biological, psychological, and sociocultural categories. Diehl and Baghurst (2016) suggested the biopsychosocial model as a framework for investigating drive for muscularity and MD. The biopsychosocial model refers to a holistic approach of conceptualizing disorders as being influenced by biological, psychological, social, and behavioral dimensions (Engel, 1977). Engel (1977) developed his model in response to inadequacies with the traditional biomedical model. He argued that psychological distress can have physical manifestations and vice versa with social and cultural environments influencing these manifestations. As suggested by Lane (2014), the biopsychosocial model is also useful for testing theories and exploring characteristics of different psychological disorders. In line with this, it has been argued that a multidisciplinary approach like the biopsychosocial framework was possibly the most exhaustive and logical way to examine MD (Olivardia, 2001). This may help identify factors influencing this condition in order to help develop prevention or treatment programs (Diehl and Baghurst, 2016). Thus, the following potential factors of influence are structured using the biopsychosocial framework, operationalizing all study variables into biological, psychological and sociocultural factors.

So far, biological factors like age or body composition have only been scarcely investigated in relation to drive for muscularity, especially not in weight-training samples. Body composition measures like body mass index (BMI) and fat-free mass index (FFMI, characterizing the muscularity of a person) were anticipated to be related to MD (Cafri et al., 2005; Grieve, 2007). Using college or community samples, little to no association with drive for muscularity was found. McCreary et al. (2006) showed that the flexed biceps circumference was able to predict muscularity-related behavior in college men, but not muscularity-related attitudes. Another study on college aged men found no connection with anthropometric measures, including body fat percentage and FFMI (Chittester and Hausenblas, 2009).

Similarly, to our knowledge, the impact of age on drive for muscularity has only scarcely been investigated. It is assumed that age, especially the time of puberty and the bodily and hormonal changes associated with it, are related to muscle building behavior. Although pubertal timing in boys is marked by general muscular growth, the engagement in body change strategies, such as exercising and use of food supplements, finds its onset here as well (Cafri et al., 2005). Therefore, this might be a time where drive for muscularity starts developing. Although there are some studies that find an increase in body dissatisfaction and/or desire to become more muscular in preadolescent and adolescents boys (McCabe et al., 2001; McCabe and Ricciardelli, 2004), there are few studies investigating a wider age range and to our knowledge none on weight-training samples. One study found that body dissatisfaction and drive for muscularity are lower in middle-aged and older man than in early adults (Bucchianeri et al., 2014), leading to the assumption, that the drive for muscularity varies over ages groups, with decreasing importance in older age.

With regard to psychological factors associated with drive for muscularity, low self-esteem has been identified by different authors in adolescent boys (McCreary and Sasse, 2000) and college aged men (Chittester and Hausenblas, 2009). Generally, associations between lower self-esteem and muscularity-related variables were found in boys (Ricciardelli and McCabe, 2003) and college students (Olivardia et al., 2004). Olivardia et al. (2004) reported a negative correlation between self-esteem and different body dissatisfaction variables, such as muscle displeasure or belittlement. More specifically, Grossbard et al. (2009) found that greater contingent self-esteem (the degree to which a positive self-image is contingent upon other factors like, e.g., appearance) was associated with greater drive for muscularity in a sample of college students. Comparing bodybuilding and weight-training men with physically active controls, it was found, that bodybuilders showed lower self-esteem than the control group (Blouin and Goldfield, 1995).

Another factor associated with drive for muscularity is body dissatisfaction (Cafri et al., 2005; Grieve, 2007). In one study, expressed dissatisfaction significantly predicted drive for muscularity in male college students (Pritchard et al., 2011). Similarly, body dissatisfaction was found to be a significant predictor for drive for muscularity in adolescent boys (Mustapic et al., 2015). More specifically, Tylka (2011) found that muscle dissatisfaction was related to muscularity-related behavior (e.g., exercising) while dissatisfaction with body fat was related to disordered eating behavior in undergraduate men. Contrariwise, a recent study reported no relation between muscle dissatisfaction and drive for muscularity-related behavior in regularly exercising men (Stratton et al., 2015). Also, it was reported that weight-training and bodybuilding men showed higher degrees of body dissatisfaction than men not engaging in these kinds of sports (Mangweth et al., 2001; Hallsworth et al., 2005).

Associated with body dissatisfaction is body ideal internalization (Grieve, 2007) of socially prescribed male body ideals, spread through mass media. In a sample of male college students, Parent and Moradi (2011) found positive interrelations between degree of internalization and drive for muscularity. Media body ideal internalization also revealed to be an important factor for drive for muscularity in adult men (Daniel and Bridges, 2010; Edwards et al., 2012) and musculature-enhancing behavior, such as weight-lifting in adolescent boys (Smolak et al., 2005). According to Stratton et al. (2015), the promotion and glorification of a muscular ideal through the media can be considered to be an essential factor for the development and maintenance of body dissatisfaction in men, comparable to the unreachably thin ideal for most women. In their study, internalization of the media body ideal was related to muscle dissatisfaction, but not directly to drive for muscularity behaviors in physically active men (Stratton et al., 2015).

Moreover, sociocultural factors such as teasing or critique by peers and parents have been suggested as related to drive for muscularity. While some studies on school-aged boys found significant correlations between the experience of being teased by parents, siblings, and peers and drive for muscularity (Schaefer and Salafia, 2014), others found no connection between peer and parental teasing and drive for muscularity (Smolak and Stein, 2006). Investigating MD symptoms in a sample of male bodybuilders, Boyda and Shevlin (2011) found a relation between childhood victimization, such as verbal, physical, and social bullying and MD. It was argued, that regular critique and emotional victimization by parents (Lamanna et al., 2010) and peers (Boyda and Shevlin, 2011) may lead to body image distortion and higher degrees of body dissatisfaction which, according to other studies (Pritchard et al., 2011; Mustapic et al., 2015), are related to drive for muscularity. More generally, it was found that negative appearance-based comments were associated with higher body dissatisfaction and higher driver for muscularity (Nowell and Ricciardelli, 2008). In a sample of bodybuilders childhood bullying experience were associated with higher scores in MD (Wolke and Sapouna, 2008).

In conclusion, most of these factors have been investigated using samples of college or community samples. A few studies also investigated weight-trainers and bodybuilders, mostly in comparison with other athlete groups, regarding symptoms of MD (Olivardia et al., 2000; Hildebrandt et al., 2006; Nieuwoudt et al., 2015). It was found, that bodybuilders reported higher rates of body dissatisfaction and drive for muscularity, as well as lower self-esteem than other groups (Blouin and Goldfield, 1995; Hallsworth et al., 2005; Hale and Smith, 2012). Other studies found that power lifters showed significantly higher rates of drive for muscularity than bodybuilders (Hale et al., 2010) and that high frequency weight-trainers had higher drive for muscularity scores than low frequency weight-trainers (Arbour et al., 2005).

The aim of this study is to extend the work conducted so far on contributing factors on drive for muscularity in (1) more detail and (2) in a weight-training sample. Instead of college students, we investigated a special population with potentially higher degrees of drive for muscularity. Bodybuilders and weight-trainers are presumed to have a higher risk to develop extreme degrees of drive for muscularity and pathological outcomes like MD (Pope et al., 1997). They generally also show higher degrees of body dissatisfaction (Mangweth et al., 2001; Hallsworth et al., 2005) and lower self-esteem (Blouin and Goldfield, 1995; Wolke and Sapouna, 2008) than other athlete or non-athlete controls. Since McCreary (2012) suggested studying drive for muscularity beyond college student populations, this strategy appeared promising in identifying potential relevant factors associated with drive for muscularity in men with high risks for body image problems.

Furthermore, we aimed to investigate factors associated with the drive for muscularity in more detail. Instead of using global assessments for body dissatisfaction and self-esteem, we examined dissatisfaction with musculature, body fat, and different facets of self-esteem simultaneously.

To this purpose, the following variables were examined in one concise model to answer the question whether (1) age, (2) FFMI, and above that (3) self-esteem facets such as performance-, physical attractiveness- or fitness-oriented self-esteem, (4) dissatisfaction with muscularity and with body fat, (5) media body ideal internalization, and (6) stressful peer experiences in childhood and adolescence can predict attitudinal and behavioral aspects of drive for muscularity in weight-training men. Attitudinal aspects represent muscularity-related attitudes like beliefs, estimates, and thoughts about muscularity, while behavioral aspects represent muscularity-related behavior like exercise and dieting (Waldorf et al., 2014). More precisely, we assumed for the biological factors, that age would negatively predict drive for muscularity while the FFMI would positively predict drive for muscularity. We assumed further, that self-esteem facets would negatively predict drive for muscularity, while body dissatisfaction, internalization and stressful peer experiences would positively predict drive for muscularity, in that higher values in each factor correlate with higher drive for muscularity. We did not expect any explicit differences between the attitudinal and behavioral aspects of drive for muscularity, except for the FFMI which we assumed to be a positive predictor for drive for muscularity-related behavior, but not attitudes.

## Materials and Methods

### Participants

Five hundred and thirty-eight participants provided consent to participate in the study. Eligibility criteria for inclusion were that the participants were male, at least 18 years of age, and that they regularly participated in weight training (operationalized as at least two times a week). Of these, 230 (42.8%) persons either failed to start the study or completed only part of it and thus were not included in the analysis. Furthermore, two participants (0.4%) under 18 years of age, 21 women (3.9%) and 15 men (2.8%) who did not train regularly had to be excluded. According to Kouri et al. (1995), the FFMI is only a meaningful measure of muscularity in individuals with lean to moderate body fat; therefore, 21 men with body fat levels of >20% (3.9%) were excluded.

The final sample consisted of 248 regularly weight-training men aged 18–51 years (*M* = 25.9, *SD* = 7.4). The sample was primarily German (66.9%) and Austrian (31.0%). Only 2.2% identified as citizens of other countries. Regarding their educational status, 34.8% of the men graduated from college, 39.1% graduated high school, and 26.1% had less than a high school education. In addition, 96.4% of the participants self-identified as heterosexual, 2.8% as bisexual, and 0.4% as homosexual, while 0.4% chose not to report their sexual orientation.

### Measures

#### Sociodemographic Measures and Exercise-Related Variables

Once informed consent was received, sociodemographic data (e.g., nationality, age, sexual orientation, educational qualification) and workout-related behavior (e.g., years and frequency of exercise, average training time, reasons for weight training) were given by the participants.

#### Fat-Free Mass Index

In contrast to the BMI (kg/m^2^), the FFMI is an objective measure of a person’s degree of muscularity for men with low or moderate percentages of body fat (Kouri et al., 1995; Pope et al., 2000b). The FFMI is calculated as follows: FFMI = (LBM 6.1 × (1.8–H))/H^2^. In this formula, LBM refers to lean body mass in kilograms, which is computed as body weight minus the percentage of body fat. H stands for height measured in meters (Pope et al., 2000a). Pope et al. (2000b) classified men with FFMI-indices of 16–17 as slightly muscular, 19–20 as typically muscular for US American or European students, 22–23 as noticeably muscular, and 25–26 as extremely muscular. FFMI-indices over 26 are very unlikely achievable without the misuse of anabolic-androgenic steroids (AASs). This classification can be used only for men with little to moderate body fat percentages, because musculature increases when body fat increases, causing one to reach FFMI-indices over 26. As a result, BMI, body fat and FFMI must be interpreted in regard to each other (Pope et al., 2000b). In order to calculate each individual’s FFMI, participants also self-reported anthropomorphic data (e.g., body height, weight, fat percentage).

#### Self-Esteem

The Multidimensional Self-Esteem Scale (MSWS, Schütz and Sellin, 2006) is an adaption of the Multidimensional Self-Concept Scale (MSCS; Fleming and Courtney, 1984) in German language. The 32 items are answered on a seven-point Likert scale from 1 (*not at all/never*) to 7 (*very much/always*) and are divided into six subscales using sum scores, namely emotional self-esteem (α = 0.89), social self-esteem – security in social contact (α = 0.86), social self-esteem – dealing with critique (α = 0.87), performance-related self-esteem (α = 0.81), self-esteem – physical attractiveness (α = 0.87) and self-esteem – fitness (α = 0.80; Schütz and Sellin, 2006). Stability was reported to be *r* = 0.46 (performance-related self-esteem) to 0.86 (and self-esteem – fitness) for the subscales. Convergent validity was investigated using correlations with the Rosenberg Self-esteem Scale and the Frankfurter Self-concept Scale for body facets, reporting correlations between *r* = 0.37 and 0.78 (Schütz and Sellin, 2006). Internal consistency for the subscales used in this study was acceptable: global self-esteem (α = 0.87), performance- (α = 0.80), physical attractiveness- (α = 0.83), and fitness-related self-esteem (α = 0.64) were used.

### Dissatisfaction with Body Fat and Muscularity

The Bodybuilder Image Grid, Scaled (BIG-S, Hildebrandt et al., 2004) is a bi-dimensional silhouette figure rating scale presenting 30 males figures with different degrees of muscularity and body fat percentages. The figures range from low to high fat on the horizontal axis and low to high muscularity on the vertical axis. Thus, it is possible to simultaneously assess current and ideal figures regarding fat and muscularity. The discrepancy between ratings of current and ideal bodies reveals a measurement of dissatisfaction with muscularity and body fat. Higher scores on either dimension represent the desire to become either more muscular or leaner. Convergent and divergent validity was demonstrated and test–retest reliability was reported to be *r* = 0.89 to 0.94 for the original version (Hildebrandt et al., 2004) and *r* = 0.71 to 0.96 for the scaled version (Santarnecchi and Dèttore, 2012).

#### Internalization of the Media Body Ideal

The German version of the Sociocultural Attitudes toward Appearance Questionnaire (SATAQ-G, Knauss et al., 2009) is an instrument used to assess the influence of sociocultural body ideals on body image. For this study, the only subscale administered was the boys’ version of the internalization of media body ideals subscale (α = 0.84). Convergent validity was reported to be acceptable (Knauss et al., 2009). The internalization subscale consists of six items rated on a five-point Likert scale from 1 (*strongly disagree*) to 5 (*strongly agree*), with higher mean scores indicating a higher internalization of a muscular, lean male body ideal.

#### Stressful Peer Experiences

To assess participants’ history of stressful peer experiences (e.g., teasing), the German questionnaire for experience of peer victimization in childhood and adolescence (FBS, Sansen et al., 2013) was used. The FBS has been shown to have good validity and test-retest reliability (*r* = 0.83; Sansen et al., 2013). This 22-item retrospective questionnaire is based on a binary response format (*experienced/ not experienced*). Construct validity was confirmed via correlations between the FBS and psychological distress and social anxiety measures (Sansen et al., 2013). Calculating the overall sum value, higher scores indicate higher levels of bullying experience.

#### Drive for Muscularity

The desire to obtain a bigger and more muscular physique was assessed with the German translation of the Drive for Muscularity Scale (DMS, Waldorf et al., 2014). The self-rating questionnaire contains 15 items. It can be divided into two subscales, *muscularity-related attitudes* (DMS attitudes, eight items) and *muscularity-related behavior* (DMS behavior, seven items). The items were rated on a six-point Likert scale from 1 (*always*) to 6 (*never*) and were reverse-coded with higher mean scores representing greater drive for muscularity. For the original version, with no distinction between the subscales, internal consistencies were reported to be good for both men (α = 0.90) and women (α = 0.83; McCreary and Sasse, 2000). In the German version internal consistency in different samples was good (global scale α = 0.89 to 0.90, muscularity-related attitudes α = 0.88 to 0.90, muscularity-related behavior α = 0.79–80; Waldorf et al., 2014). Retest-reliability was also good (global scale *r* = 0.95, muscularity-related attitudes *r* = 0.92, muscularity-related behavior *r* = 0.96) and validity was confirmed using a correlation (*r* = 0.81) with the Male Body Attitudes Scale (Waldorf et al., 2014). In the present sample, Cronbach’s alpha for the global scale was 0.89, for the muscularity-related attitudes 0.90, and for the muscularity-related behavior 0.75.

### Procedure

Participants were recruited through advertisement in online panels for the German-speaking weight-training and bodybuilding community. German, Austrian, and Swiss panels were provided with a web link to the survey, which took approximately 30 min to complete. Before starting the online survey, participants were informed about the study and its purpose. All participants took part on a voluntary and anonymous basis and were treated in accordance with the declaration of Helsinki. After completion of the survey, which was provided only in German language, participants were given the possibility to take part in a lottery with the chance of winning one of two 50 € vouchers for dietary supplements as an incentive.

### Data Management and Analysis

Prior to analyses, scores on the predictor variables and the DMS were examined for outliers. We z-transformed and checked all outcome variables for values out of an absolute variation of three standard deviations. No unacceptable values were being found^1^. All assumptions for regression analysis have been met, specifically no multicollinearity, the normality of the dependent variables, no autocorrelations, and homoscedasticity of the residuals.

Using a hierarchical forced entry method, three multiple linear regression analyses were conducted. All variables were investigated regarding their relation with (1) drive for muscularity, in addition to each of the (2) muscularity-related attitudes, and (3) muscularity-related behavior subscales. Since there is only marginal evidence for the association with biological factors, such as age or FFMI (Cafri et al., 2005), these variables were added separately. Thus, all three regression analysis were conducted with two steps each, (1) one for the biological factors and (2) one for the psychological and sociocultural factors.

## Results

### Weight-Training Related Outcomes

As shown in **Table 1**, respondents stated exercising a mean of 3.96 times per week (*SD* = 7.37) for 79.87 min per session (*SD* = 23.93). The mean of years of exercise was 5.05 (*SD* = 5.45). Average BMI indicated slight overweight status (*M* = 25.57, *SD* = 2.93); however, body fat percentage (*M* = 13.36, *SD* = 3.49) was in the lower healthy range for men between 20 and 39 years of age (Gallagher et al., 2000). Compared to a study of the general population of Austria (*M* = 25.9), this sample had a comparable BMI value (*M* = 25.57), but a much lower body fat percentage (*M* = 13.36 vs. *M* = 22.3; Elmadfa et al., 2012). This shows that the BMI is only partly useful within a population of highly muscular individuals, and other body composition measures are needed.

**Table 1:** Descriptive statistics for demographic and anthropometric variables.

Variable	Mean	*SD*	Minimum	Maximum
Age	25.87	7.37	18	51
Weight (kg)	83.56	11.16	60	135
Height (m)	1.81	0.07	1.60	2.03
BMI	25.57	2.93	18.22	41.21
FFMI	22.09	2.54	16.52	36.20
Frequency weight training per week	3.96	7.37	2	7
Minutes weight training	79.87	23.93	3	180
Years weight training	5.05	5.45	0.25	34

*BMI, body mass index; FFMI, fat-free mass index.*

The mean value for the FFMI was 22.09 (*SD* = 2.54). Based on the classification of Pope et al. (2001), 6.4% of the men described their musculature as below average, 30.6% as average, and 62.8% as above average. 8.9% of the sample disclosed former or current use of illegal, physically enhancing supplements (e.g., AAS) to become more muscular. The majority pursued the goal of gaining more muscle mass (84.7%), improving physical appearance (79.4%), increasing well-being (74.6%), promoting health (72.6%), and increasing strength (69.8%). Further aims included the reduction of body fat (43.5%), performance improvement in other sport activities (20.6%), participation in bodybuilding competitions (15.7%), and weight loss (8.1%). In addition, 88.3% of all participants indicated dieting in order to achieve lean muscularity.

### Biological Factors

As presented in **Table 2**, along with the semi-partial correlations (the factors unique contribution to the outcome), age was the strongest negative predictor (i.e., the predictor greatest in magnitude if the regression coefficients) for aspects of drive for muscularity, while FFMI revealed the second-strongest positive connection with drive for muscularity-related behavior. On the other hand, FFMI did not predict muscularity-related attitudes. Both factors accounted for 9–14% of variance in drive for muscularity and its related attitudes and behaviors.

**Table 2:** Results from multiple linear regression models predicting the facets of drive for muscularity (DMS).

Drive for muscularity		DMS mean score			DMS attitudes			DMS behavior		
		β	*t*	*r*_a(bc)_	β	*t*	*r*_a(bc)_	β	*t*	*r*_a(bc)_
Age		-0.36	-5.81^∗∗∗^	-0.35	-0.35	-5.74^∗∗∗^	-0.34	-0.29	-4.68^∗∗∗^	-0.28
FFMI		0.07	1.11	0.07	-0.07	-1.12	-0.07	0.23	3.70^∗∗∗^	0.22
Self-esteem – physical attractiveness		-0.22	-2.93^∗∗^	-0.12	-0.20	-2.84^∗∗^	-0.11	-0.19	-2.13^∗^	-0.11
Self-esteem – fitness		-0.20	-3.34^∗∗^	-0.14	-0.17	-3.05^∗∗^	-0.12	-0.19	-2.61^∗^	-0.13
Performance-related self-esteem		-0.04	-0.60	-0.03	-0.03	-0.44	-0.02	-0.05	-0.59	-0.03
Global self-esteem		0.10	0.90	0.04	-0.01	-0.08	-0.00	0.21	1.62	0.08
Dissatisfaction with muscularity		0.15	3.23^∗∗^	0.13	0.21	4.81^∗∗∗^	0.19	0.04	0.63	0.03
Dissatisfaction with body fat		0.04	0.83	0.03	0.02	0.50	0.02	0.05	0.91	0.05
Internalization media body ideals		0.47	10.34^∗∗∗^	0.42	0.41	9.39^∗∗∗^	0.37	0.45	8.13^∗∗∗^	0.41
Stressful peer experiences		-0.02	-0.45	-0.02	-0.01	-0.30	-0.01	-0.03	-0.46	-0.02

Model fit	Step 1	*F*(2,245) = 16.969^∗∗∗^			*F*(2,245) = 20.327^∗∗∗^			*F*(2,245) = 14.113^∗∗∗^		
	Step 2	*F*(10,237) = 35.898^∗∗∗^			*F*(10,237) = 41.777^∗∗∗^			*F*(10,237) = 16.544^∗∗∗^		
*R*^2^	Step 1	0.12			0.14			0.10		
*R*^2^	Step 2	0.60			0.64			0.41		
Δ*R*^2^		0.48			0.50			0.31		

*r_a(b c)_, semi-partial correlation, ^∗^p < 0.05, ^∗∗^p < 0.01, ^∗∗∗^p < 0.001, FFMI, fat-free mass index; Step 1, age and FFMI; Step 2, psychological and sociocultural factors.*

### Psychological Factors

Regarding the psychological factors both body-related self-esteem facets, physical attractiveness and fitness significantly predicted overall drive for muscularity. They also predicted muscularity-related attitudes and muscularity-related behavior. In all cases they were negative predictors, in that lower self-esteem facets predicted higher values of drive for muscularity and its subscales.

Global and performance-related self-esteem failed to predict drive for muscularity, although on a correlational level, significant relations have been found (global self-esteem: *r* = -0.26 to -0.52, *p* < 0.001; performance related self-esteem: *r* = -0.16 to -0.31, *p* < 0.01).

Dissatisfaction with muscularity significantly predicted muscularity-related attitudes but not muscularity-related behavior in that higher levels of dissatisfaction with muscularity predicted higher values of muscularity-related attitudes. Dissatisfaction with body fat on the other hand, was no significant predictor for drive for muscularity, although it was significantly related to it on a correlational level (*r* = -0.18 to -0.22, *p* < 0.01).

### Sociocultural Factors

The strongest positive predictor for overall drive for muscularity, muscularity-related attitudes, and behavior was internalization of the media body ideal. Stressful social experiences were not significantly predicting drive for muscularity, nor one of its subscales. It showed only small significant correlations to drive for muscularity (*r* = 0.15) and its attitudinal aspects (*r* = 0.18).

In conclusion, psychological and sociocultural variables accounted for 60% of variance in drive for muscularity, 64% of the variance in attitudinal, and 41% of the variance in behavioral aspects of drive for muscularity. Therefore, dissatisfaction with muscularity, body-related self-esteem and internalization of media body ideals enhanced the model significantly, leading to a 31% change in *R*^2^ in behavior, 50% in attitudes, and 48% in total drive for muscularity.

## Discussion

Using a biopsychosocial model as a framework, the aim of this study was to investigate the associations of biological, psychological, and sociocultural variables with drive for muscularity in weight-training men. As predicted, these variables explained a significant amount of the variance in drive for muscularity and its subscales. In support of the hypotheses, internalization of media body ideals, dissatisfaction with muscularity, and aspects of self-esteem, which include physical attractiveness and fitness, as well as age and FFMI, each demonstrated significant predictors for weight-training men’s drive for muscularity. On the contrary, dissatisfaction with body fat, global self-esteem, performance-related self-esteem, and stressful peer experience were not significantly associated with drive for muscularity.

### Internalization of Media Body Ideals

Consistent with previous research on school and college samples (Daniel and Bridges, 2010; Stratton et al., 2015; Edwards et al., 2016), internalization of media body ideals was the strongest predictor of drive for muscularity and its related attitudes and behavior in weight-training men. Mass media play a key role in distributing an unrealistic muscular and lean male body image, which becomes the body ideal for many boys and men trying to achieve this goal by any means (Smolak et al., 2005; Cafri et al., 2006; Parent and Moradi, 2011; Readdy et al., 2011). To our knowledge, there are no studies explicitly investigating the impact on internalization of media body ideals in weight-training samples so far, but it can be assumed, that this sample shows higher degrees of internalization, since they might also be closer to this ideal than the usual samples of school boys and college or community men. Although this is only of descriptive value, we have found one study (Parent and Moradi, 2011) also using the internalization subscale of the SATAQ, showing a quite lower mean (*M* = 1.75, *SD* = 0.71) in a sample of college men than our sample (*M* = 2.88, *SD* = 0.93). Since increased levels of internalization were associated with increased drive for muscularity, it appears useful to develop prevention approaches for the weight-training community helping to reveal the potential harm that comes with the belief in, and massive consumption of, unrealistic body ideals.

### Age

As a scarcely investigated factor, age showed to be a strong negative predictor for drive for muscularity. Increased age was related to lower global, attitudinal, and behavioral facets of drive for muscularity. It is possible that, over time, other aspects of life, such as career, financial resources, or family become more important than physical appearance, resulting in a reduction of drive for muscularity. Alternatively, the desired level of muscularity might be attained and the initial need be satisfied. The relation of age and drive for muscularity could be moderated by different degrees of internalization of media body ideals. Current data support Grieve’s (2007) notion that the muscular ideal appears to be very salient for adolescents and college men, but might be less important to older samples. This is consistent with Stratton et al. (2015), who found men older than college age to be less dissatisfied by bodily comparison. Self-esteem and body dissatisfaction could also be interacting with age, since it has been shown, that in adult life with higher age, both, self-esteem (Robins et al., 2002) and body satisfaction (Tiggemann and McCourt, 2013) are rising. Future research, especially longitudinal studies, are required to further examine the relationship between age and drive for muscularity, as well as developmental and hormonal influences in younger age.

### Self-Esteem

In line with other findings (McCreary and Sasse, 2000; Chittester and Hausenblas, 2009) we found relations between certain facets of low self-esteem and drive for muscularity. While fitness-related self-esteem represents to what extent individuals are content with their athletic and coordination skills, physical attractiveness-related self-esteem symbolizes the satisfaction with one’s appearance and a person’s confidence to be attractive (Schütz and Sellin, 2006). Both showed a negative relation with drive for muscularity. Fitness-related self-esteem was related to all facets of drive for muscularity. Physical attractiveness related self-esteem was associated only with global and attitudinal, but not behavioral aspects. This could result from peoples’ assumptions that physical attractiveness is often associated with facial attractiveness (Currie and Little, 2009), so that there might be no expectation that exercising could have an influence on physical attractiveness-related self-esteem. Global self-esteem and performance-related self-esteem, although significantly correlated to drive for muscularity, did not reach statistical significance in the regression analysis, pointing to the vital influence of body-related aspects of self-esteem in opposition to more general self-esteem variables. Although, it has been found that weight-training and bodybuilding samples report higher self-esteem than controls (Pickett et al., 2005), body related self-esteem seems to be important for the desire to become more and more muscular, which is of significance especially in populations with higher risk for MD.

### Body Dissatisfaction

To our knowledge, this is one of the few existing examinations of dissatisfaction with muscularity as well as with body fat in relation to drive for muscularity in weight-training men. According to the literature (Blouin and Goldfield, 1995; Hildebrandt et al., 2004; Olivardia et al., 2004), it was assumed that both aspects of body dissatisfaction would be associated with drive for muscularity. Even though stronger dissatisfaction with muscularity was linked to global and cognitive aspects of drive for muscularity, it showed no relation with behavioral facets. This is in line with findings of Stratton et al. (2015), but contradicting those of Karazsia and Crowther (2010) and Tylka (2011), who found that dissatisfaction with muscularity significantly predicted muscularity-related behavior in college and community men. A reason for this could be that cognitive mechanisms and actual behavior are very distinct features. Just as not all women who are dissatisfied with their weight start dieting, not all men who are dissatisfied with their muscularity start increasing their muscularity-related behavior, especially when this behavior is already above average. Future research should investigate these two facets separately, especially regarding extreme forms of drive for muscularity.

Dissatisfaction with body fat failed to predict drive for muscularity. According to Tylka (2011), dissatisfaction with musculature is related to muscularity-related behavior, while dissatisfaction with body fat is associated with disordered eating behavior. Since body dissatisfaction in general and drive for muscularity have been found to be distinct concepts (Bergeron and Tylka, 2007), it seems plausible that drive for muscularity can be understood as a strategy to reduce dissatisfaction with muscularity. Thus, dissatisfaction with body fat, related to different behaviors of coping (e.g., dieting, cardio workout) is unrelated to drive for muscularity.

### Fat-Free Mass Index

Even though some researchers have presented the advantages of the FFMI over the BMI (Cafri et al., 2005), to our knowledge, only two studies (using college samples) have systematically analyzed the FFMI in relation to drive for muscularity (McCreary et al., 2006; Chittester and Hausenblas, 2009). In contrast to Chittester and Hausenblas (2009) who did not detect any relations, we found an association of FFMI with muscularity-related behavior, but not with attitudes. This is partly in line with findings of McCreary et al. (2006). It appears that higher muscle mass is related to more muscularity-related behaviors. Thus, current results suggest that muscle growth relates to the behavioral, but not to attitudinal features of drive for muscularity.

Future research needs to focus on a standardized operationalization and interpretation of the FFMI, since no clear instructions have been published and existing interpretations of the FFMI are inconsistent. Clearly, replication with the use of skin calipers or hydrostatic weighing (Cafri et al., 2005) instead of self-reported body composition measures is recommended.

### Stressful Peer Experience

Contrary to the hypotheses, stressful social experiences in childhood and adolescence were not associated with drive for muscularity, although a significant small positive correlation was found. These results are inconsistent with other findings (Nowell and Ricciardelli, 2008; Schaefer and Salafia, 2014). Thus, the assumption that men taking up intense exercise do so in order to make up for earlier stressful experience (Wolke and Sapouna, 2008) and use weight training as a coping strategy (Boyda and Shevlin, 2011) can be questioned.

Yet, it is possible that stressful social experience serve as a moderator or mediator. Lamanna et al. (2010) argued that regular critique and emotional victimization through parents leads to higher degrees of body dissatisfaction, which again, is associated with drive for muscularity. Also, exercise as a coping strategy for more recent stressful experiences might be of more relevance than for those from the past. Therefore, including stressful social experience through peers, parents, and partners, especially related to body shape, might be useful in future research.

### Strengths and Limitations

A number of strengths can be named for the current study. Since the majority of research in this field uses school or college samples, the recruitment of a sufficiently large sample of weight-training men can be considered to be more meaningful for those persons at high risk for body image problems. Also, the investigation of biological, psychological and sociocultural factors simultaneously, using a biopsychosocial framework, allowing assumptions on different degrees of importance for drive for muscularity, can be useful. Further, investigating biological factors, such as the FFMI and age separately, provides important directions for future research. Especially, the effect of age, along with hormonal influences should be investigated on a longitudinal level, since changes in its relation to drive for muscularity can be assumed as being non-linear.

The current study also has a number of limitations. As described regarding the influence of age, a longitudinal approach could lead to more valid findings. A cross-sectional design, as used in this study, limits inference with regard to the temporal order in which the factors may operate. Also, online studies rely on self-reported measures, which are especially prone to biases in regard to body measures. Although, bodybuilders and weight-trainers can be assumed to have more precise knowledge about their bodies than the average person, on-site measuring would be preferable and should be used in future studies.

Another limitation concerns the homogeneity of the sample. First, since we used a weight-training sample, no generalization on the general population can be drawn. Since it is usually weight-training and bodybuilding men who are at risk of pathological outcomes of extreme drive for muscularity, the results might still be valuable for prevention and treatment in this special population. Second, results are only generalizable for men with relatively low body fat percentages, since the FFMI was only interpretable when body fat percentage was below 20%. Third, although our sample is, on average, 5 years older than the usual college sample, no conclusions can be drawn for weight-training men in older age categories. Moreover, the vast majority of the current sample is from Western, industrialized, German-speaking countries and generalization is only viable for these populations. Furthermore, there was a very low percentage of homosexual or bisexual men in this sample (3.2%). Since homosexuality is discussed as a risk factor for eating disorders in men (Russell and Keel, 2002; Freeman, 2005), it would be interesting to investigate its relation to drive for muscularity further.

## Conclusion

This study aimed at further investigating factors associated with drive for muscularity in weight-training men, using a biopsychosocial model as a frame. Our current findings confirmed most of the hypothesized factors as being associated with global drive for muscularity and muscularity-related attitudes and behaviors. Internalization of media body ideals presented the highest predictive value for drive for muscularity. This indicates that the drive for muscularity, similar to the drive for thinness in women, seems to be a problem of societies in which highly unrealistic body ideals are advertised and idealized. As a consequence, education on, and advertisement of, body ideals based on health and well-being, instead of thinness or leanness and muscularity, should be pursued in order to promote health instead of appearance.

## Ethics Statement

The study was conducted in accordance with the principles of the Declaration of Helsinki and with institutional guidelines of the School of Psychology, University of Vienna. Furthermore, it followed the Guidelines of the ethical conduct proposed by the American Psychological Association. According to the institutional guidelines of the University of Vienna, Austria (http://satzung.univie.ac.at/ethikkommission-der-universitaet-wien/), approval by an ethics committee was not necessary because the study did not affect the physical or psychological integrity, the right for privacy, or other personal rights or interests (see § 2(1)). All participants gave informed consent on the first page of the online survey (prior to the actual start of the study) after having received a written description of the study. Data collection was anonymous and no harmful procedures were used. Furthermore, participation was voluntarily and no remuneration for participation was given.

## Author Contributions

CS and LR designed and conducted the study in consultation with KH-F. CS and LR analyzed the data with assistance and contributions from MV and KH-F. CS drafted the manuscript with contributions from LR, MV, and KH-F. All authors read and approved the final manuscript.

## Conflict of Interest Statement

The authors declare that the research was conducted in the absence of any commercial or financial relationships that could be construed as a potential conflict of interest.

## Footnotes

1 One participant reported spending just 3 min on weight-training per session and thus, constituted an outlier. However, removal of this participant’s data did not impact the results. Therefore, his data were retained.
